# Supplementary material for: Improved Cultivation and Isolation of Diverse Endophytic Bacteria Inhabiting Dendrobium Roots by Using Simply Modified Agar Media
Source: Microbiol Spectr. 2022 Oct 27;10(6):e02238-22. doi: 10.1128/spectrum.02238-22 (PMC9769524; doi:10.1128/spectrum.02238-22)
Supplement: Supplemental file 1 — Fig. S1 and Table S1. Download spectrum.02238-22-s0001.pdf, PDF file, 0.09 MB [file spectrum.02238-22-s0001.pdf]

Supplementary Figure and Table

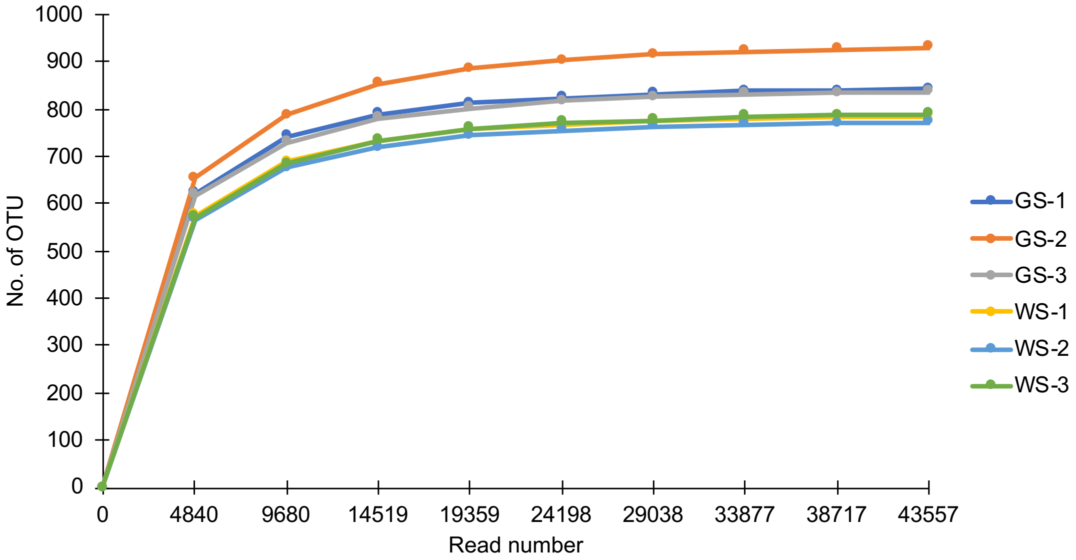

Supplementary Figure S1. Rarefaction curves for endophytic bacterial operational taxonomic units (OTUs) from each *Dendrobium* sample. GS: green stem-*Dendrobium* root and WS: white stem-*Dendrobium* root.

Supplementary Table S1 Statistics of Miseq amplicon sequencing of endophytic bacterial communities in the *Dendrobium* roots

Root sample	No. of raw reads	No. of reads after Qiime2 process	No. of OTUs
GS-1	183219	45725	843
GS-2	200196	51299	934
GS-3	179852	43557	837
WS-1	189484	46691	787
WS-2	183392	43614	772
WS-3	196759	50086	792

GS: green stem-*Dendrobium* root and WS: white stem-*Dendrobium* root.
